# Supplementary material for: A community pharmacist-led smoking cessation intervention using a smartphone app (PharmQuit): A randomized controlled trial
Source: PLoS One. 2022 Mar 29;17(3):e0265483. doi: 10.1371/journal.pone.0265483 (PMC8963551; doi:10.1371/journal.pone.0265483)
Supplement: S2 File — (PDF) [file pone.0265483.s002.pdf]

A community pharmacist-led smoking cessation intervention using a smartphone app (PharmQuit): A randomized controlled trial

Research Protocol

Introduction

Smoking is a major cause of premature deaths worldwide [1]. The death rate from any cause among current smokers was about three times that of those who had never smoked [2-3]. Smoking cessation before the age of 40 reduces the risk of death associated with continued smoking by about 90 percent [2]. The goal of the Thai government to reduce the prevalence of smoking by 15 percent by the year 2025is an ongoing challenge for all public health workers [2].

Pharmacists play a pivotal role in providing smoking cessation services. Many systematic reviews have shown that pharmacist-led interventions resulted in better abstinence rates in smokers [4-7], and may also be cost-effective [6, 8]. However, some studies have not shown clear benefits of pharmacy personnel-delivered Nicotine Replacement Therapy (NRT) interventions [6, 9]. Medications for cessation showed an abstinence rate of 26.59 percent at 6 months and 19.90 percent at 12 months [10]. A smoking cessation service, provided in a hospital, for 94 smokers showed a 6-month quit rate of 31.9 percent and a 12-month quit rate of 17 percent. The loss to follow-up rate was 75.5 percent [11]. In a smoking cessation service which started with 23 community pharmacies, catering for 205 smokers at the start, had only 2 participating community pharmacies after 6 months [12]. Pharmacists reported difficulties with follow-up associated with participants' relapse[13]. Clearly, there were issues in communication between community pharmacists and smokers.

A web-based Electronic health informatics-based intervention showed a 6-month abstinence rate of 17 percent [14]. A Thai telephone counseling service, called Quitline, showed a 6-month quit rate of 33.1 percent [15]. Current evidence shows benefits of mobile phone-based smoking cessation interventions on long-term outcomes (RR 1.71, 95% CI 1.47 to 1.99, over 9,000 participants) [16]. Various mobile apps are already available to help smokers quit, such as: SmartQuit, DistractMe, mCM, SmokeFree, Craving to Quit, and REQ-Mobile [17]. A systematic review showed only two out of 50 apps had scientific and professional support [18], and most apps omitted referrals to Quitline and recommendations for medicine [19-20]. Participants did not stay in the program and over 50% of the study participants dropped out due to a variety of factors, including mobile phone or operating system/app incompatibility and difficulties with quitting [21]. The quality of smoking cessation apps according to the US Public Health Service's Clinical Practice Guideline for Treating Tobacco Use and Dependence was low [18, 22-23] and most apps were not customized to users' needs [17].

The Community Pharmacy Association Thailand and Community Pharmacy Network for Smoke-free Thai Society support training for community pharmacists to provide smoking cessation services with follow-up on day 0, 7, 14, 30, 60, and 120. Some medications for cessation such as NRT, herbal lozenges/spray, and nortriptyline are also dispensed under the

supervision of community pharmacists. However, low smoker retention rates, low quit rates, and the weakness of electronic health interventions were challenges. PharmQuit, a mobile application, was designed to fill these gaps by incorporating pharmacists and User Experience (UX). Using human computer interaction (HCI), PharmQuit focuses on positive emotions to ensure the desired experience [24].

Current evidence shows benefits of mobile phone-based smoking cessation interventions on long-term outcomes [25]. A few studies of smoking cessation apps have been conducted in community pharmacies and evaluated for short term (8 weeks) outcomes [26-27]. Aim of the study was to develop a smartphone app, PharmQuit, and evaluate the effectiveness of pharmacist-led quit-smoking smartphone app on abstinence rate, number of cigarettes smoked per day, carbon monoxide level, adherence to the smoking cessation program, and satisfaction with the app.

Research question

How much an app could increase quit rate when use it with smoking cessation program in community pharmacies?

Objective of the research

1. To develop a smart phone app for helping smokers to quit smoking (PharmQuit)
2. To compare effectiveness of smoking cessation program by community pharmacy (usual care) and usual care with PharmQuit app by using these measures
    - 2.1 number of cigarette smoked per day
    - 2.2 quit rate, point abstinence rate, continuous abstinence rate
    - 2.3 exhaled carbon monoxide
    - 2.4 adherence rate
    - 2.5 satisfaction to PharmQuit

Research scope

This study design was prospective randomized control trial with a control group. The study area was covered 4 provinces under the National Health Security Office region 7 Khon Kaen; Khon Kaen, Maha Sarakham, Roi-et, and Kalasin. The study time was firstly plan to be between October, 2016 and September 2017, however, the recruitment was performed between July 30, 2017 and August 28, 2018. T The study completed in January 2019. This trial was registered retrospectively in the Thai Clinical Trials Registry: TCTR20200925004 on September 25, 2020.

Conceptual framework

Figure 1 Conceptual framework

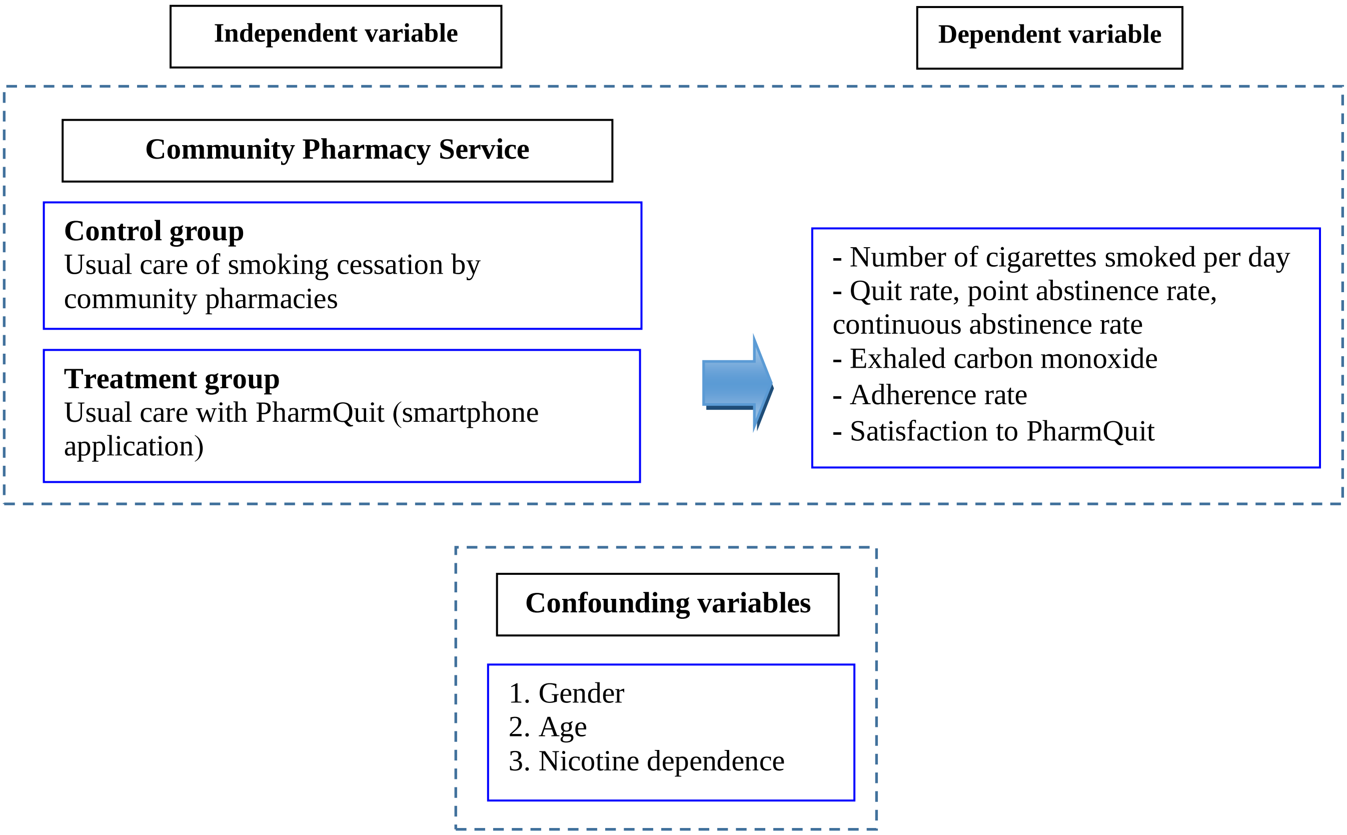

Outcome measures were shown in Figure 1 as the dependent variables; quit rate, point abstinence rate, continuous abstinence rate exhaled carbon monoxide (CO), adherence rate, and satisfaction to PharmQuit. The three confounding factors (gender, age, nicotine dependence) had been controlled [28].

Expected benefits

1. Collaboration with multidisciplinary in smoking cessation service by smartphone application
2. It could be basic information for the National Health Security Office region 7 Khon Kaen to support smoking cessation service.

Definitions

1. Smartphone application is an application developed to use with smartphone for smoking cessation.
2. Service in the control group is a usual care provided by a pharmacist following 5As (Ask, Advise, Assess, Assist, Arrange follow-up) as recommended by a practice guide for smoking cessation year 2012 [29].
3. Service in the treatment group is a usual care with smartphone app (PharmQuit)
4. Effectiveness is to compare outcomes between the control and treatment groups in measures of number of cigarettes smoked per day, quit rate, point abstinence rate,

continuous abstinence rate exhaled carbon monoxide; CO adherence rate, and satisfaction to PharmQuit.

5. Quit smoking means continuous abstinence for 6 months [30].
6. Point prevalence abstinence means number of smokers reported with no smoking for 7 days.
7. Continuous abstinence means that smokers stop smoking after received counseling and/or medications continuously until the next follow-up visit.
8. Adherence rate means follow-up at Day 7, 14, 30, 60, 120, and 180.

Methodology

This study design was a prospective randomized control trial with a control group. It aimed to compare the usual care of smoking cessation by community pharmacies with the usual care with an application. An application was developed for helping smokers and pharmacists to work together to quit smoking.

Population and sample

Population: smokers who came to get smoking cessation service from community pharmacies in the area of Health National Security Office region 7 (4 provinces: Khon Kaen, Maha Sarakham, Roiet, and Kalasin)

Sample: smokers who came to get smoking cessation service from community pharmacies, willing to join the research during October 2016-September 2017

Inclusion criteria

1. Age at least 18 years
2. Smoking one cigarette per day for at least 1 month
3. Ready to quit smoking
4. Be able to answer question, do self-recording or having a smartphone and be able to use application
5. Willing to join the research and be followed-up

Exclusion criteria

1. Having pregnancy or breast feeding
2. Having underlying disease or chronic diseases which required special care
3. Being with other cessation programs or methods
4. Being unable to contact consecutively for 3 times or quit to participate the research

Sample calculation

$$n_1 = \frac{(Z_\alpha+Z_\beta)^2(PQ)(r+1)}{(P_1-P_0)^2 r}$$

$$\bar{P} = \frac{(P_1+rP_0)}{(1+r)} \quad \text{และ} \quad r = \frac{n_0}{n_1}$$

When:

n_1 = number of sample in the treatment group

n_0 = number of sample in the control group
r = proportion of the control and treatment groups
Z_α = standard value of Z with type I error at $\alpha/2$
Z_β = standard value of Z with type II error at β
$\bar{P}$ = proportion of average outcomes from 2 groups
$\bar{Q} = 1 - \bar{P}$
P_1 = proportion of quit rate in the treatment group
P_0 = proportion of quit rate in the control group

In order to compare between 2 groups, the number of sample is set to be equal, so r = 1 at the confident level of 95%. The power of test is at 80%.

$Z_\alpha = 1.96$, $Z_\beta = 0.84$
P_1 = proportion of quit rate in the treatment group
P_0 = proportion of quit rate in the control group

From a study in Perth, Australia, 8 community pharmacies showed quit rate 13.8% in the treatment group and 1.3% in the control group at month 6 (χ^2 1=9.0, P=.003). This confirmed the result by carbonmonoxide exhale test [31].

Calculation:

$$n1 = \frac{(1.96+0.84)^2(0.076*0.925)(1+1)}{(0.138-0.013)^2(1)}$$

$$= \frac{(2.8)^2(0.07)(2)}{(0.125)^2}$$

$$= \frac{(7.84)(0.07)(2)}{0.016}$$

$$= 69$$

Adding 15% for drop-out rate, 11 people, the total calculated sample will be 80 people per group.

Tools

1. A1-A2 service form (Appendix A)
2. A4 service form (Appendix B)
3. A5 service form (Appendix C)
4. Structure development of smartphone applications (PharmQuit) (Appendix D)
5. Satisfaction questionnaire to PharmQuit (Appendix E)
    - A 25-item questionnaire was developed for the satisfaction assessment. The questionnaire had six parts: objective, scope, interactive features, design, beauty, and self-efficacy.
    -
6. Self-report form (Appendix F)
7. Subject information sheet and consent form (Appendix G)
8. Carbon monoxide machine
9. Smoking cessation service guideline for 5As

Methods

Part 1: Application development for smoking cessation (PharmQuit)

Set the aims and framework of PharmQuit application to serve the need of smokers who are ready to quit smoking. If smokers are not ready to quit smoking, messages are sent to motivate for quitting. When smokers join the cessation program, motivations, goal for quitting, and reminders to keep being abstinent will be assessed. Barriers will be identified to prevent exposures. The service model creates interaction between the application and users for fun. There may be rewards for users who use application regularly and/or pass each step to achieve success. Passing each step in the program will increase self-confidence to quit smoking. Information including adverse events will be available. Every day follow-up is to encourage to quit smoking as shown in Figure 2.

Figure 2 Application concept for PharmQuit

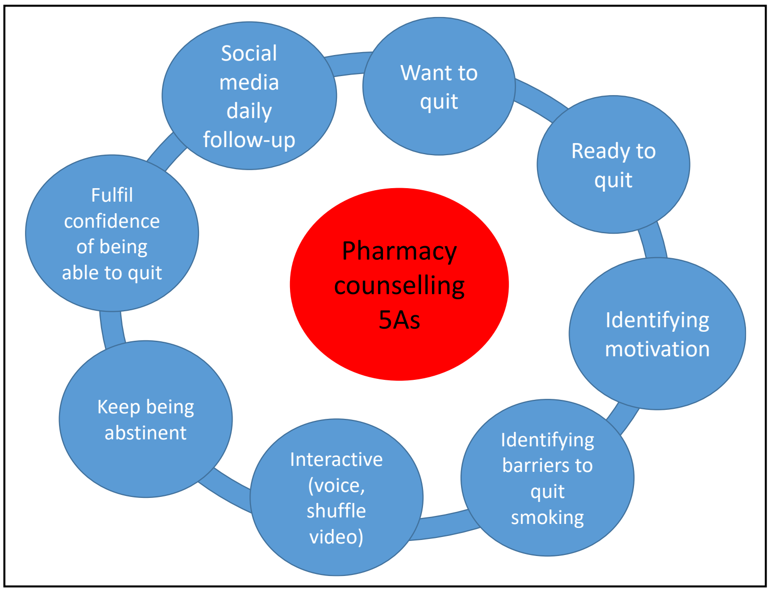

Application development

1. Reviewing research publications related to features of applications both in Thailand and other countries. It was to study and use information from previous study for making a new app for cessation with easy steps, uncomplicated, good looking for increasing quit rate [28, 32, 33].
2. Arranging 3 meetings with four experts for smoking cessation counseling. It was to interview for commonly use questions, sequences of questions in real practice. Then a researcher synthesized information and presented the systematic approach questions in a flow chart. This flow chart was used to talk with programmers (appendix D).
3. Structure of app was to work with smoking cessation service from community pharmacies. Application is to use for encouraging, giving information during craving, tracking progress in cessation. The users had to download app and register to the app with inputting personal information, smoking behavior, intention and motivation to quit smoking, assessment for nicotine addiction, and quit smoking. Everyday an encouraging message presented on the

app. A reminding message presented on the app for a user to keep a record of smoking or no smoking, adverse events and resolutions as shown in Figure 2. The user could use the app for encouraging themselves, searching information to deal with adverse events.

4. After app was completely developed, the pilot test was performed in 30 students in order to assess for easiness, beauty, execution, stability of the app, and satisfaction to the app.

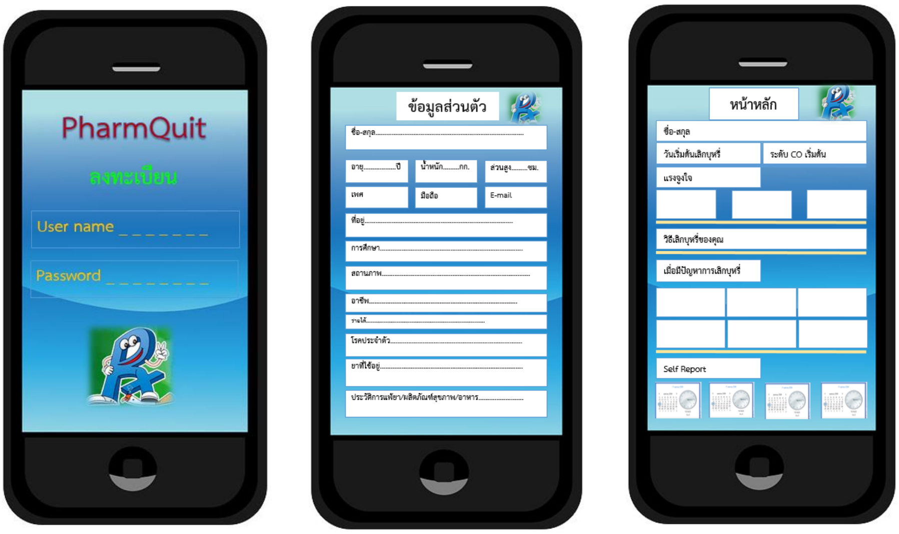

Figure 2 Screen shots of PharmQuit app

Part 2: Evaluation the effectiveness of smoking cessation service between the control and treatment groups

1. Preparing the tools and testing for validity by three experts in tool development and smoking cessation counselling.
2. Applying for ethics to Mahasarakham University Ethics committee.
3. Coordinating with community pharmacies in the area of National Health Security Office region 7 Khon Kaen.
4. Arranging a meeting for training counseling for smoking cessation and using application PharmQuit. A research file contained smoking cessation counseling, how to assess exhale CO, and PharmQuit manual.
5. Recruiting smokers from community pharmacies and assigning them to either the control group or the treatment group. Stratify random sampling with gender, age, and nicotine addiction was planned as shown in Figure 3. Age group is classified according to the Thai statistics for smokers. Then simple random sampling was used to assign smokers to either to the control or treatment groups. A represented to the treatment group, and B represented the control group as shown in Table 1.
6. Table 1 was printed out for community pharmacists who joined the research.
7. Pharmacists registered smokers to the smoking cessation service, and assigned smokers to either group according to the randomization table. The control group received usual smoking cessation service. The treatment group received usual smoking cessation service with PharmQuit app as shown in Figure 4.

Figure 3 Stratified randomization sampling

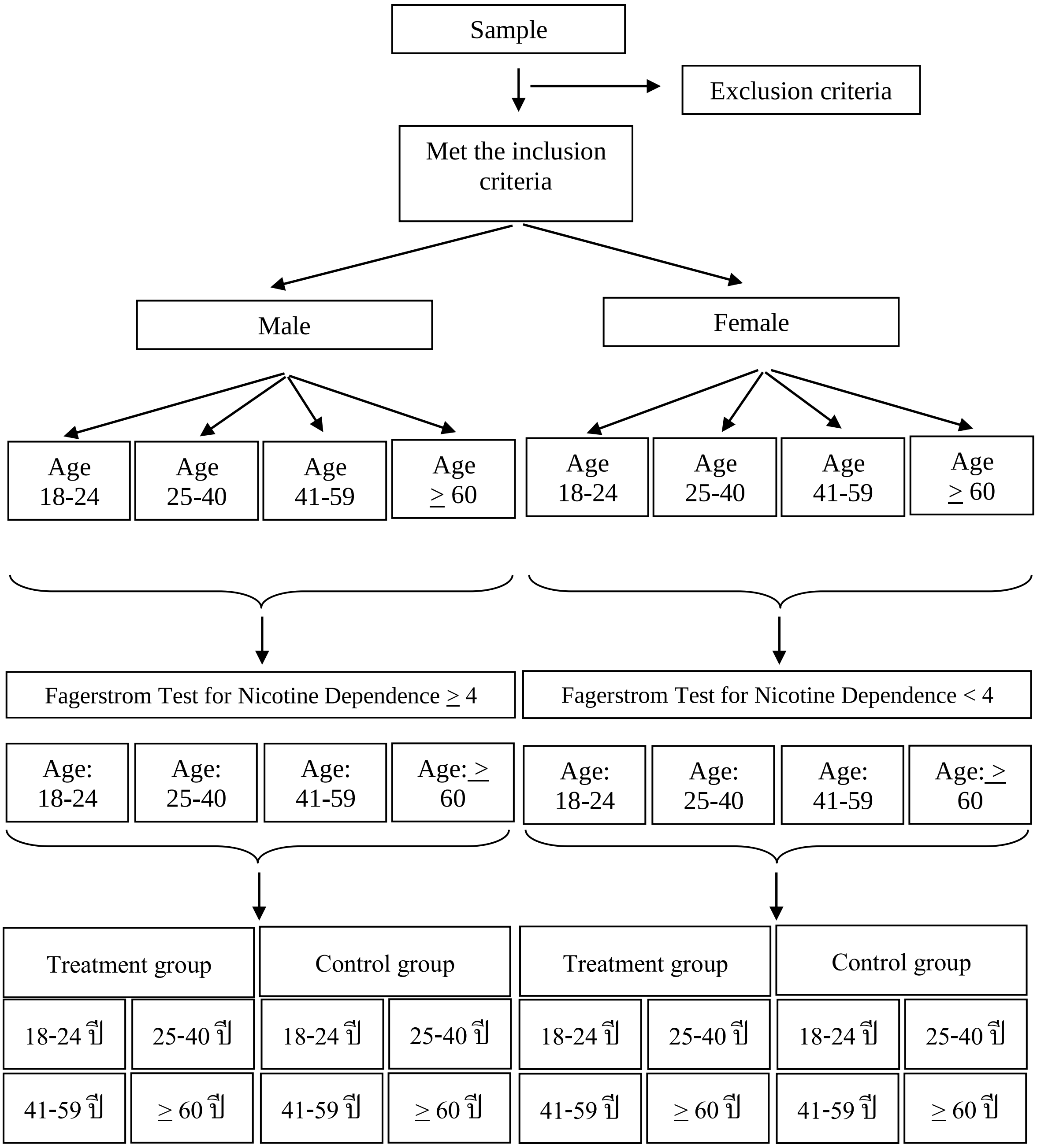

Table 1 Simple random sampling table to allocate participants the control or treatment groups for one community pharmacy

Gender	Male								Female							
Age (years)	18-24		25-40		41-59		> 60		18-24		25-40		41-59		> 60	
FGST	≥4	<4	≥4	<4	≥4	<4	≥4	<4	≥4	<4	≥4	<4	≥4	<4	≥4	<4
No 1	A	B	A	B	A	B	B	A	A	B	B	B	A	B	A	B
No 2	B	A	B	A	B	A	A	B	B	A	A	A	B	A	B	A
No 3	A	B	A	B	A	B	A	A	B	A	B	A	A	A	B	A
No 4	B	A	B	A	B	A	B	B	A	B	A	B	B	B	A	B
No 5	A	B	A	A	A	B	B	B	A	A	B	B	A	B	A	A
No 6	B	A	B	B	B	A	A	A	B	B	A	A	B	A	B	B
No 7	B	B	B	B	A	B	B	A	A	B	A	A	B	B	A	B
No 8	A	A	A	A	B	A	A	B	B	A	B	B	A	A	B	A
No 9	A	A	B	B	A	A	B	B	A	B	A	B	A	B	B	B
No 10	B	B	A	A	B	B	A	A	B	A	B	A	B	A	A	A
No 11	A	A	B	B	A	A	A	B	B	B	B	B	B	A	B	A
No 12	B	B	A	A	B	B	B	A	A	A	A	A	A	B	A	B
No 13	B	B	B	B	A	A	B	B	A	A	B	B	B	A	A	B
No 14	A	A	A	A	B	B	A	A	B	B	A	A	A	B	B	A
No 15	A	B	B	A	A	B	A	A	B	A	A	B	B	A	A	A
No 16	B	A	A	B	B	A	B	B	A	B	B	A	A	B	B	B
No 17	B	B	B	B	A	A	B	B	B	A	B	A	A	B	A	B
No 18	A	A	A	A	B	B	A	A	A	B	A	B	B	A	B	A
No 19	A	A	B	A	B	A	B	B	A	A	A	B	B	A	B	A
No 20	B	B	A	B	A	B	A	A	B	B	B	A	A	B	A	B
No 21	B	A	B	B	B	B	A	B	B	B	A	A	B	B	B	A
No 22	A	B	A	A	A	A	B	A	A	A	B	B	A	A	A	B
No 23	B	A	B	B	A	B	A	A	B	B	B	B	B	B	B	A
No 24	A	B	A	A	B	A	B	B	A	A	A	A	A	A	A	B
No 25	B	A	A	B	A	A	B	A	B	B	B	B	B	B	A	B
No 26	A	B	B	A	B	B	A	B	A	A	A	A	A	A	B	A
No 27	A	A	B	A	A	B	A	B	A	B	A	A	A	B	B	B
No 28	B	B	A	B	B	A	B	A	B	A	B	B	B	A	A	A
No 29	A	B	B	A	B	B	A	A	B	B	A	B	B	A	A	B
No 30	B	A	A	B	A	A	B	B	A	A	B	A	A	B	B	A

Figure 4 Outcome measures and following-up between the control and treatment groups

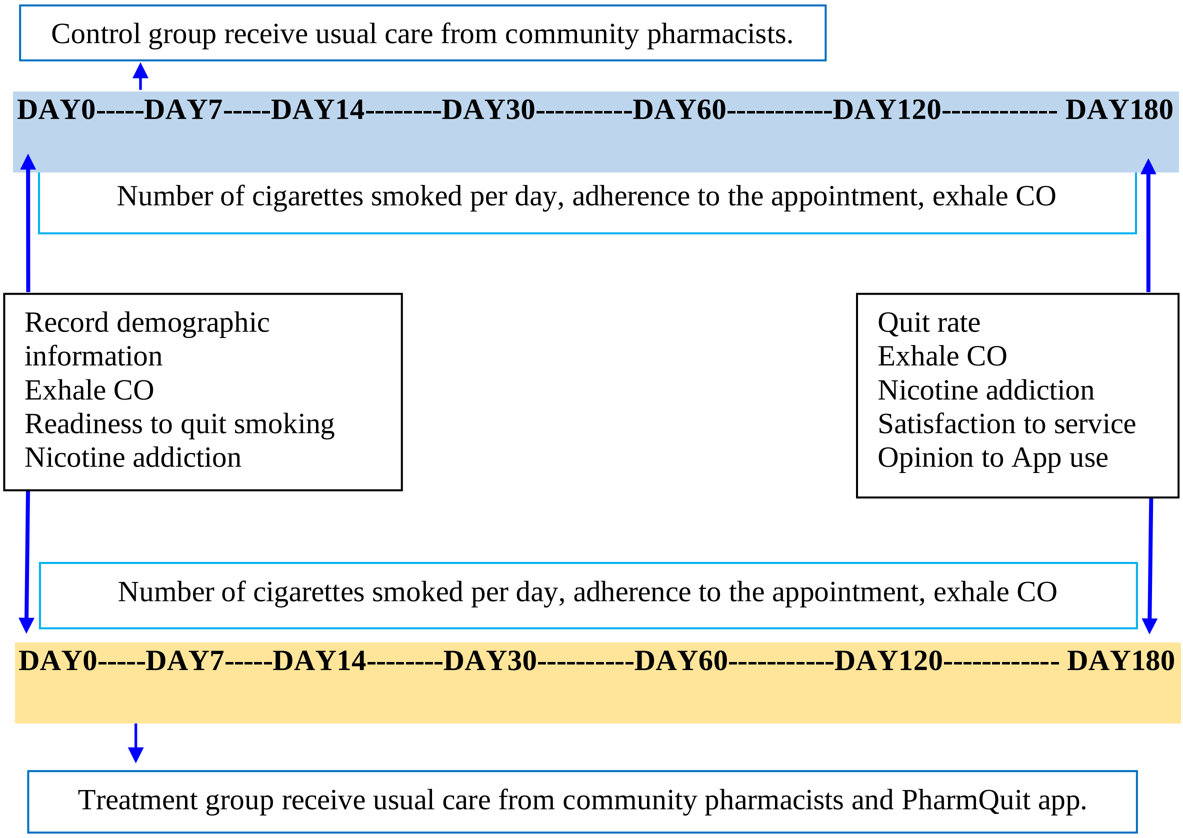

The control group

1. Pharmacists/researchers collected demographic information including name, age, gender, body weight, height, body mass index, heart rate, exhaled CO, education, marital status, career, income, underlying disease, allergic history, and home address.
2. Smoking cessation service by a community pharmacy
   1) A community pharmacist provided counseling by using 5As principle (Ask, Advise, Assess, Assist, Arrange follow-up). A community pharmacy made an appropriate plan for individual smoker including identifying motivation to quit smoking; emphasized the importance of quit, no return, and quit right now; modifying lifestyle; planning to deal with craving.
   2) A community pharmacist gave advice and worked together with smokers for a proper lifestyle modification for each smoker such as training themselves when thinking of smoking, avoiding aggravating factors, doing something else when needing smoking.
   3) When the nicotine addiction level (Fagerstorm test for nicotine dependence; FTND) was lower than 4 scores, a pharmacist provides counseling to quit smoking following 5Ds, STAR. When the FTND was 4 or higher, together with smoking at least 10 cigarettes per day, or had a history of failure to quit smoking, medications were dispensed e.g. sodium nitrate 0.5% mouth wash, *Vernonia*

cinerea lozenges, and herbal medicine as appropriate. Contraindications were checked before dispensing. The pharmacists counseled smokers on how to use the medication: drug name, dose, regimen, administration, duration of therapy, adverse effects, and disposal of nicotine gum. Pharmacists dispensed the medications following the smoking cessation practice guidelines of Thailand.

3. Day 0 at a community pharmacy, first outcome measures were number of cigarettes smoked per day, exhaled CO, FTND, blood pressure, bodyweight, height.
4. Following up in Day 7, 14, 30, 60, 120, and 180 by telephone and/or visits at community pharmacy. If participants did not come, a community pharmacist would call the participants or send message.
5. Day 7, 14, 30, 60, and 120, outcome measures were number of cigarettes smoked per day, exhaled CO, craving symptoms, adverse events from medication use, adherence to an appointment.
6. Day 180, outcome measures were outcome measures are number of cigarettes smoked per day, exhaled CO, FTND, craving, adverse events, blood pressure, body weight, height, and satisfaction to service.

Treatment group

1. Pharmacists/researchers collected demographic information including name, age, gender, body weight, height, body mass index, heart rate, exhaled CO, education, marital status, career, income, underlying disease, allergic history, and home address.
2. Community pharmacists provided smoking cessation with PharmQuit app.
    - A community pharmacist provided counseling by using 5As principle (Ask, Advise, Assess, Assist, Arrange follow-up). A community pharmacy made an appropriate plan for individual smoker including identifying motivation to quit smoking; emphasized the importance of quit, no return, and quit right now; modifying lifestyle; planning to deal with craving.
    - A community pharmacist gave advice and worked together with smokers for a proper lifestyle modification for each smoker such as training themselves when thinking of smoking, avoiding aggravating factors, doing something else when needing smoking.
    - When the nicotine addiction level (Fagerstorm test for nicotine dependence; FTND) was lower than 4 scores, a pharmacist provided counseling to quit smoking following 5Ds, STAR. When the FTND was 4 or higher, together with smoking at least 10 cigarettes per day, or had a history of failure to quit smoking, medications were dispensed e.g. sodium nitrate 0.5% mouth wash, *Vernonia cinerea* lozenges, and herbal medicine as appropriate. Contraindications were checked before dispensing. The pharmacists counseled smokers on how to use the medication: drug name, dose, regimen, administration, duration of therapy, adverse effects, and disposal of nicotine gum. Pharmacists dispensed the medications following the smoking cessation practice guidelines of Thailand.
    - Pharmacists registered PharmQuit app for participants and taught them how to use it. PharmQuit would help a pharmacist in counseling and following up participants with features of self-report of craving symptoms, adverse drug reactions, and how to deal with adverse events
3. Day 0 at a community pharmacy, first outcome measures were number of cigarettes smoked per day, exhaled CO, FTND, blood pressure, bodyweight, height.

4. Following up in Day 7, 14, 30, 60, 120, and 180 by telephone and/or visits at community pharmacy. If participants did not come, a community pharmacist could call the participants or send message.
7. Day 7, 14, 30, 60, and 120, outcome measures were number of cigarettes smoked per day, exhaled CO, craving symptoms, adverse events from medication use, adherence to an appointment, and difficulties in using PharmQuit.
8. Day 180, outcome measures were outcome measures are number of cigarettes smoked per day, exhaled CO, FTND, craving, adverse events, blood pressure, body weight, height, satisfaction to service and opinion to PharmQuit.

Statistical analysis

1. Descriptive statistics were used to calculate mean (SD) and percentage for quit rate, point abstinence rate, continuous abstinence rate, number of cigarette smoked per day, exhaled CO, adherence rate, and satisfaction to PharmQuit.
2. Comparison of quit rate, point abstinence rate, continuous abstinence rate between groups, Chi-square test was used. Comparison of satisfaction to PharmQuit between groups, student t test was used when data showed normal distribution. If the data was not normally distributed, Mann-Whitney U test was performed.
3. Logistic regression used to compare quit rate within group and between groups at Day 7, 14, 30, 60, 120, 180.
4. Intention-to-treat was applied to the analysis. Data for participants with missing data were assumed to be the same as their last visit information. All tests were two-sided and alpha was set to 5%.

Ethics consideration

The study protocol, consent forms, and tools received ethical approval by Mahasarakham University (ID: 033/2559).

References

1. Taylor AL, Bettcher DW. WHO Framework Conventional on Tobacco Control: a global "good" for public health. *Bull World Health Organ* 2000;78:920-9.
2. Jha P, Ramasundarahettige C, Landsman V, Landsman V, Rostron B, Thun M, et al. 21st-Century Hazards of Smoking and Benefits of Cessation in the United States. *N Engl J Med* 2013;368:341-350.
3. Thun MJ, Carter BD, Feskanich D, Freedman ND, Prentice R, Lopez AD, et al. 50-Year Trends in Smoking-Related Mortality in the United States. *N Engl J Med* 2013;368:351–364.
4. Saba M, Diep J, Saini B, Dhippayom T. Meta-analysis of the effectiveness of smoking cessation interventions in commucity pharmacy. *J Clin Pharm Ther* 2014;39:240-247.
5. Brown TJ, Todd A, O'Malley C, Moore HJ, Husband AK, Bambra C, et al. Community pharmacy-delivered interventions for public health priorities: A systematic review of interventions for alcohol reduction, smoking cessation and weight management, including meta-analysis for smoking cessation. *BMJ open*. 2016;6:e009828.
6. Brett K, Yenug SST, Ford C. Pharmacist-led interventions for tobacco smoking cessation: a review of clinical effectiveness and cost-effectiveness. CADTH Rapid Response Report: Summary with Critical Appraisal. Ottawa (ON): *CADTH*; 2019 Sep 9. [online] https://europepmc.org/article/NBK/NBK549529 (accessed 13 April 2021)

7. O'Reilly E, Frederick E, Palmer E. Models for pharmacist-delivered tobacco cessation services: a systematic review. *J Am Pharm Assoc* 2019;59:42-752.
8. Cantor SB, Deshmukh AA, Luca NS, Nogueras-Gonzalez GM, Rajan T, Prokhorov AV. Cost-effectiveness analysis of smoking-cessation counseling training for physicians and pharmacists. *Addict Behav* 2015;45:79-86.
9. Mdege ND, Chindove S. Effectiveness of tobacco use cessation interventions delivered by pharmacy personnel: a systematic review. *Res Social Adm Pharm* 2014;10: 21-44.
10. Rosen LJ, Galili T, Kott J, Goodman M, Freedman LS. Diminishing benefit of smoking cessation medications during the first year: a meta-analysis of randomized controlled trials. *Addiction* 2018;113:805-816.
11. Bussaratid S, Siripaiboonkij A. Study of Smoking Cessation Rate at Smoking Cessation Clinic, Siriraj Hospital, Thailand. *J Psychiatr Assoc Thailand* 2012;57:305-312.
12. Bunditanukul K, Bunditanukul W, Chalongsuk, R. Effectiveness of smoking cessation program by the community pharmacist in BANGKOK. *Thai Bull of Pharm Sci* 2014;9:1-17.
13. Shen X, Bachyrycz A, Anderson JR, Tinker D, Raisch DW. Improving the effectiveness of pharmacist-assisted tobacco cessation: a study of participant- and pharmacy-specific differences in quit rates. *Ann Phar* 2015;49:303-310.
14. Shahab L, McEwen A. Online support for smoking cessation: a systematic review of the literature. *Addiction* 2019;104:1792-1804.
15. Meeyai A, Yunibhand J, Punkrajang P, Pitayarangsarit S. An evaluation of usage patterns, effectiveness and cost of the national smoking cessation quitline in Thailand. *Tobacco Control* 2015;24:481-488.
16. Whittaker R, McRobbie H, Bullen C, Borland R, Rodgers A, Gu Y. Mobile phone-based interventions for smoking cessation. *Cochrane Database Syst Rev* 2012;11: CD006611. doi: 10.1002/14651858.CD006611.pub3.
17. Hoeppner BB, Hoeppner SS, Seaboyer L, Schick MR, Wu GW, Bergman BG, et al. How smart are smartphone apps for smoking cessation? A content analysis. *Nicotine Tob Res* 2015;18:1025-1031.
18. Haskins BL, Lesperance D, Gibbons P, Boudreaux ED. A systematic review of smartphone applications for smoking cessation. *TBM* 2017;7:292-299.
19. Abroms LC, Lee Westmaas J, Bontemps-Joanes J, Ramani R, Mellerson J. A content analysis of popular smartphone apps for smoking cessation. *AM J Prev Med* 2013;45:732-736.
20. Ubhi HK, Michie S, Kotz D, van Schayck OCP, Selladurai A, West R. Characterising smoking cessation smartphone applications in terms of behavior change techniques, engagement and ease-of-use features. *TBM* 2016; 6:410-417.
21. Godon JS, Armin J, D Hingle M, Giacobbi P Jr, Cunningham JK, Jahonson T, et al. Development and evaluation of the See Me Smoke-Free multi-behavioral mHealth app for women smokers. *TBM* 2017; 7:172-184.
22. Abroms LC, Padmanabhan N, Thaweethai L, Phillips T. iPhone apps for smoking cessation: a content analysis. *Am J Prev Med* 2011;40:279-285.
23. Jacobs MA, Cobb CO, Abroms L, Graham AL. Facebook apps for smoking cessation: a review of content and adherence to evidence-based guidelines. *JMIR* 2014;16:e 205. http://dx.doi.org/10.2196/jmir3491.
24. Hassenzahl M, Tractinsky N. User experience - a research agenda. *Behav Inf Technol* 2006;25:91-97.
25. Whittaker R, McRobbie H, Bullen C, Borland R, Rodgers A, Gu Y. Mobile phone-based interventions for smoking cessation. *Cochrane Database Syst Rev*. 2012;11. DOI: 10.1002/14651858.CD006611.pub3.
26. Herbec A, Brown J, Shahab L, West R, Raupach T. Pragmatic randomized trial of a smartphone app (NRT2Quit) to improve effectiveness of nicotine replacement therapy in a quit attempt by improving medication adherence: results of prematurely terminated study. *Trials.* 2019;20:547.

27. Iacoviello BM, Steinerman JR, Klein DB, Silver TL, Berger AG, Luo SX, Schork NJ. Clickotine, a personalized smartphone app for smoking cessation: initial evaluation. *JMIR Mhealth Uhealth.* 2017;5(4): e56.
28. BinDhim NF, McGeechan K and Trevena L. Assessing the effect of an interactive decision-aid smartphone smoking cessation application (app) on quit rates: a double-blind automated randomized control trial protocol. *BMJ Open*. 2014; 4: e005371.
29. Rungruanghiranya S, Sunthorntham S. Smoking cessation practice guideline in Thailand. Bangkok: Health Promotion Foundation; 2012.
30. SRNT Subcommittee on Biochemical Verification. Biochemical verification on tobacco use and cessation. *Nicotine Tob Res* 2002; 4(2): 149-59.
31. Burford O, Jiwa M, Carter O, Parsons R, Hendrie D. Internet-Based Photoaging Within Australian Pharmacies to Promote Smoking Cessation: Randomized Controlled Trial. *J Med Internet Res.* 2010 Mar; 15(3): e64.
32. Heffner JL, Vilardaga R, Mercer LD, Kientz JA, Bricker JB. Feature-level Analysis of a Novel Smartphone Application for Smoking Cessation. *Am J Drug Alcohol Abuse*. 2015 Jan; 41(1): 68-73.
33. Thai Health Promotion Foundation. “Thai Rai Kwan” application for smoking cessation 2016. Available at: http://www.thaihealth.or.th/Content/30688-"ไทยไร้ควัน"%20แอปพลิเคชั่นเลิกบุหรี่.html
