# Supplementary material for: A community pharmacist-led smoking cessation intervention using a smartphone app (PharmQuit): A randomized controlled trial
Source: PLoS One. 2022 Mar 29;17(3):e0265483. doi: 10.1371/journal.pone.0265483 (PMC8963551; doi:10.1371/journal.pone.0265483)
Supplement: S3 File — (PDF) [file pone.0265483.s003.pdf]

Questionnaire: Satisfaction with PharmQuit

Objective

The purpose of this questionnaire is to assess your satisfaction with PharmQuit and help make improvements to the program.

Section1: General information

Please mark the appropriate box [ ] and fill in necessary information.

1. Gender [ ]1. Male [ ]2. Female
2. Age ………………years
3. Marital status [ ]1. Single [ ]2. Married [ ]3. Widowed [ ]4. Divorced
4. Education [ ]1. Did not complete High school [ ]2. High school
   [ ]3. College degree lower than Bachelor [ ]4. Bachelor Degree
   [ ]5. Graduate Degree [ ]6. Others (please specify) ………
5. Career [ ]1. Student [ ]2. Agriculture [ ]3. Government employee/state enterprise
   [ ]4. Business owner [ ]5. Hired employee
   [ ]6. Other (please specify) …………………………
6. Income (baht/month) [ ]1. <5,000 [ ]2. 5,000-9,999 [ ]3. 10,000-14999
   [ ]4. 15,000-29,999 [ ]5. ≥ 30,000
7. Underlying disease [ ]1. None [ ]2. Diabetes [ ]3. Hypertension [ ]4. Heart disease
   [ ]5. Asthma [ ]6. Obesity [ ]7. Depression
   [ ]8. COPD [ ]9. Other (please specify) ………………….
8. Do you currently smoke?
   [ ]1. I stopped smoking for ……………months………..days after joining the program
   [ ]2. I still smoke………….cigarettes/day. I used to smoke………..cigarettes/day

Section 2: Satisfaction with PharmQuit

Please mark your satisfaction with the service received by checking the most appropriate box. Five is very satisfied, four is satisfied, three is neutral, two is unsatisfied, and one is very unsatisfied.

PharmQuit	Satisfaction score				
	5	4	3	2	1
Dimension 1: Objective to quit smoking					
1. You are satisfied with PharmQuit in helping you to keep service schedules.					
2. You are satisfied with the progress feature.					
3. You are satisfied with the encouragement received					
4. You are satisfied with question and answer section.					
5. You are satisfied that PharmQuit has helped you quit or reduce the number of cigarettes smoked.					

PharmQuit	Satisfaction score				
	5	4	3	2	1
Dimension 2: Scope of application					
6. You are satisfied with the number of functions.					
7. You are satisfied with interactive functions between a pharmacist and other smokers.					
8. You are satisfied with the ease of inputting your personal information.					
9. You are satisfied with the privacy of your information.					
Dimension 3: Format and interactive between PharmQuit and the user					
10. You are satisfied with the daily encouraging messages and reminders.					
11. You are satisfied with the response speed of the application.					
12. You are satisfied with humorous and interesting features.					
13. You are satisfied with the challenging and attractive interactive features.					
Dimension 4: Design					
14. You are satisfied with characteristics of the app.					
15. You are satisfied with the amount of information on each screen.					
16. You are satisfied with the sequence of each group of functions.					
17. You are satisfied with the convenience and ease of use of PharmQuit.					
Dimension 5: Appearance					
18. You are satisfied with attractiveness and usability of the app.					
19. You are satisfied with the font and background color.					
20. You are satisfied with the font and font size.					
21. You are satisfied with beautiful and attractive pictures used.					
Which parts of the app did you like?<br><br>………<br>…….<br>Which parts should be improved?<br><br>………<br>…….					

แบบสอบถามความพึงพอใจต่อการใช้แอพพลิเคชั่น Pharm Quit และการเข้ารับบริการเลิกบุหรี่

วัตถุประสงค์ :

เพื่อสอบถามความพึงพอใจของท่านต่อการใช้งานแอพพลิเคชั่น Pharm Quit และการเข้ารับบริการเลิกบุหรี่ สำหรับการนำไปใช้ปรับปรุงระบบให้สมบูรณ์และตรงกับความต้องการของผู้รับบริการมากยิ่งขึ้น

ส่วนที่ 1 ข้อมูลทั่วไป

โปรดทำเครื่องหมาย ✓ ลงใน [ ] และกรอกข้อมูลที่ตรงกับความเป็นจริงของตัวท่าน

1. เพศ [ ]1. ชาย [ ]2. หญิง

2. อายุ ....ปี

3. สถานะภาพสมรส [ ]1. โสด [ ]2. สมรส [ ]3. หม้าย [ ]4. หย่า

4. ระดับการศึกษา [ ]1. ต่ำกว่ามัธยมศึกษา [ ]2. มัธยมศึกษา [ ]3. อนุปริญญา
[ ]4. ปริญญาตรี [ ]5. สูงกว่าปริญญาตรี [ ]6. อื่นๆ(ระบุ)....................

5. อาชีพ [ ]1. นักเรียน/นักศึกษา [ ]2. เกษตรกรรม [ ]3. ข้าราชการ/รัฐวิสาหกิจ
[ ]4. ค้าขาย/ธุรกิจส่วนตัว [ ]5. ลูกจ้างเอกชน/พนักงานบริษัท [ ]6. อื่นๆ(ระบุ)......

6. รายได้ (บาท/เดือน) [ ]1. < 5,000 [ ]2. 5,000-9,999 [ ]3. 10,000-14,999
[ ]4. 15,000-29,999 [ ]5. ≥30,000

7. โรคประจำตัว (ตอบได้มากกว่า 1 ข้อ)
[ ]1. ไม่มีโรคประจำตัว [ ]2. โรคเบาหวาน [ ]3. โรคความดันโลหิตสูง
[ ]4. โรคหัวใจ [ ]5. โรคหืด [ ]6. ภาวะอ้วนลงพุง
[ ]7. โรคซึมเศร้า [ ]8. โรคปอดอุดกั้นเรื้อรัง [ ]9. อื่นๆ(ระบุ)......................

8.ปัจจุบันท่านสูบบุหรี่หรือไม่
[ ]1. หยุดสูบแล้วเป็นเวลา............เดือน............วัน หลังจากเข้ารับบริการ
[ ]2. ยังสูบอยู่............มวน/วัน จากเดิมเคยสูบ............มวน/วัน

ส่วนที่ 2 ความพึงพอใจต่อหน้าที่แอพพลิเคชั่น Pharm Quit

โปรดระบุความพึงพอใจต่อการใช้งานแอพพลิเคชั่น PharmQuit ของท่าน โดยทำเครื่องหมาย ✓ ในช่องคะแนนที่ท่านพิจารณาแล้วตรงกับความพึงพอใจของท่านมากที่สุดเพียงช่องเดียว

ระดับความพึงพอใจ แบ่งเป็น 5 ระดับ ได้แก่ 5 หมายถึง มากที่สุด 4 หมายถึง มาก 3 หมายถึง ปานกลาง 2 หมายถึง น้อย 1 หมายถึง น้อยที่สุด

แอพพลิเคชั่น Pharm Quit	ระดับความพึงพอใจ				
	5	4	3	2	1
1. ท่านพอใจที่แอพพลิเคชั่นช่วยให้ท่านมารับบริการตรงตามนัดหมาย					
2. ท่านพอใจต่อการแสดงความก้าวหน้าในการเลิกสูบบุหรี่					
3. ท่านพอใจที่ได้รับกำลังใจในการเลิกบุหรี่จากการใช้แอพพลิเคชั่น					
4. ท่านพอใจต่อข้อมูลในส่วนการถามตอบที่ตรงกับความต้องการของท่าน					
5. ท่านพอใจที่แอพพลิเคชั่นสามารถช่วยท่านให้ลดหรือเลิกบุหรี่ได้					
6. ท่านพอใจต่อฟังก์ชั่นของแอพพลิเคชั่นที่มีครบถ้วนตามต้องการ					
7. ท่านพอใจต่อระบบโต้ตอบระหว่างผู้ให้บริการและผู้เลิกบุหรี่ท่านอื่น					
8. ท่านพอใจต่อความสามารถในการเพิ่มข้อมูลส่วนตัว					
9. ท่านพอใจต่อความเป็นส่วนตัวของข้อมูล					
10. ท่านพอใจที่มีการส่งข้อความให้กำลังใจและแจ้งเตือนทุกวัน					
11. ท่านพอใจในความเร็วของการตอบสนองของแอพพลิเคชั่น					
12. ท่านพอใจที่แอพพลิเคชั่นใช้งานสนุก ไม่น่าเบื่อ					
13. ท่านพอใจการตอบโต้ของแอพพลิเคชั่นที่ท้าทาย น่าทดลอง					
14. ท่านพอใจรูปลักษณ์ของแอพพลิเคชั่นตรงกับความต้องการ					
15. ท่านพอใจต่อการแบ่งหมวดหมู่ของแต่ละหน้าจอมีความเหมาะสม					
16. ท่านพอใจต่อการจัดลำดับแต่ละหมวดหมู่ของฟังก์ชั่น					
17. ท่านพอใจในการใช้งานที่มีความสะดวกและง่าย					
18. ท่านพอใจที่แอพพลิเคชั่นมีความสวยงาม และน่าใช้					
19. ท่านพอใจต่อสีของตัวอักษรและพื้นหลัง					
20. ท่านพอใจต่อรูปแบบและขนาดตัวอักษรที่ง่ายต่อการอ่าน					
21. ท่านพอใจรูปภาพที่ใช้มีความสวยงามและดึงดูดการใช้งาน					

ส่วนใดของแอพพลิเคชั่นที่ท่านชอบ

....

ส่วนใดของแอพพลิเคชั่นที่ท่านเห็นว่าควรปรับปรุง

....
