# Supplementary material for: A community pharmacist-led smoking cessation intervention using a smartphone app (PharmQuit): A randomized controlled trial
Source: PLoS One. 2022 Mar 29;17(3):e0265483. doi: 10.1371/journal.pone.0265483 (PMC8963551; doi:10.1371/journal.pone.0265483)
Supplement: Attachment — Submitted filename: Response to reviewers.docx [file pone.0265483.s004.docx]

**Response to Reviewers**

| **No.** | **Requirements** |  |
| --- | --- | --- |
| 6 | **Comments to the Author**  1. Is the manuscript technically sound, and do the data support the conclusions?  The manuscript must describe a technically sound piece of scientific research with data that supports the conclusions. Experiments must have been conducted rigorously, with appropriate controls, replication, and sample sizes. The conclusions must be drawn appropriately based on the data presented.  Reviewer #1: Partly  Reviewer #2: Partly | Thank you, we rewrote the conclusion. (Line 37-39) |
|  | 2. Has the statistical analysis been performed appropriately and rigorously?  Reviewer #1: No  Reviewer #2: I Don't Know | We rewrote this section. (Line 168-186) |
|  | 3. Have the authors made all data underlying the findings in their manuscript fully available?  The PLOS Data policy requires authors to make all data underlying the findings described in their manuscript fully available without restriction, with rare exception (please refer to the Data Availability Statement in the manuscript PDF file). The data should be provided as part of the manuscript or its supporting information, or deposited to a public repository. For example, in addition to summary statistics, the data points behind means, medians and variance measures should be available. If there are restrictions on publicly sharing data—e.g. participant privacy or use of data from a third party—those must be specified.  Reviewer #1: No  Reviewer #2: Yes | We added more supporting information. |
|  | 4. Is the manuscript presented in an intelligible fashion and written in standard English?  PLOS ONE does not copyedit accepted manuscripts, so the language in submitted articles must be clear, correct, and unambiguous. Any typographical or grammatical errors should be corrected at revision, so please note any specific errors here.  Reviewer #1: No  Reviewer #2: Yes | We used Editage to check our manuscript. |
|  | 5. Review Comments to the Author  Please use the space provided to explain your answers to the questions above. You may also include additional comments for the author, including concerns about dual publication, research ethics, or publication ethics. (Please upload your review as an attachment if it exceeds 20,000 characters) |  |
|  | **Reviewer #1:** This is a very interesting randomised controlled studying evaluating the efficacy of an app called PharmQuit to help with smoking cessation in Thailand. Using Pharmacists to carry out the interventions.  Well done to team for carrying out such important research.  They are some comments worth mentioning for the authors attention.  1) Line 103, can the aims be clear in terms of what the primary objectives, secondary objectives are. | We rewrote in the introduction part (Line 80-84) and in outcome part (Line 153-159). |
|  | 2) Under design, include that it was multi-centre/site since recruitment occurred in 7 community pharmacies in 3 provinces | We added in Line 24 and Line 88. |
|  | 3) The sample size calculation is incomplete, what was the quit rate (i.e the assumed quit rate together with what the ‘improvement’ was planned to be? | We explained more in Line 107-111. |
|  | 4) In addition if the quit rate was to be estimated at 6 months follow-up, this implies that 6 month is the primary timepoint. This needs to be clear especially when mentioning the aims. | We rewrote in Line 154-159. |
|  | 5) Randomization allocation ratio, I assume it was 1:1, this needs to be explicitly stated. | We added in Line 116. |
|  | 6) Although this an open-label study, allocation concealment needs to be mentioned and explicitly stated who was not blinded. | We added in Line 117. |
|  | 7) For the randomization procedure of the pharmacist checking the randomisation list, what was the assurance there was no selection bias. Was an external check/validation carried out. For an example how do you know say the next group allocation was control group but then the Pharmacist then allocated the person to intervention? | We added in Line 119-121. |
|  | 8) It would benefit clarity of the manuscript if there was a separate heading “Outcomes”. That way to make it explicit that outcome is binary, misleading to use rate as this implies a count when infact it’s a logistic model that has been fitted. Its also confusing, as it looks like there were two primary outcomes i.e. quit rate and number of cigarettes smoked per day <http://journals.plos.org/plosone/s/data-availability#loc-unacceptable-data-access-restrictions>. <http://journals.plos.org/plosone/s/data-availability#loc-unacceptable-data-access-restrictions>.And whether this is a co-primary end point, this needs to elaborated in more detail. | We put a heading of outcomes and rewrote this section in Line 153-167. |
|  | 9) Also how was the satisfaction scored? I.e. total score for each dimension?  And with such small numbers of 14 people completing the questionnaire, is it best to give a median instead with range?  It would help if the Title for Table 6 also includes that this was restricted to intervention group only. You probably also need a ‘missing’ summary as well. | We added explanation in Line 161-167.  Thank you, we presented in median and range, and edited the title of Table 8 (new). We added in a summary of missing in Line 261-263. |
|  | 11) “We randomized a total of 156 participants to the intervention group or the control group.” Best if this also includes numbers randomised to each arm. | We added in Line 188. |
|  | 12) In terms of the statistical analysis, the data structure was repeated measurements of some outcomes, should this have incorporated a mixed effects model approach. | We reanalyzed with mixed models and stated in Line 183. |
|  | 13) how was missing data handled? | We rewrote in Line 174-177. |
|  | 14) Any statistical analysis plan written? and presumably signed off prior to any data analysis. | We rewrote in Line 178-186. |
|  | **Items with CONSORT checklist**  **1) Outcomes (Item 6a)**  Completely defined pre-specified primary outcome measure including how and when it was assessed  Is it clear (1) what the primary outcome is (usually the one used in the sample size calculation), (2) how it was measured (if relevant; e.g. which score used), (3) at what time point, and (4) what the analysis metric was (e.g. change from baseline, final value)?  Not adequately defined. Primary timepoint not specified as data is being collected at different timepoints. | We rewrote the outcomes in Line 153-167. |
|  | **2) Sample size (Item 7a)**  How sample size was determined  Is there a clear description of how the sample size was determined, including (1) the estimated outcomes in each group; (2) the α (type I) error level; (3) the statistical power (or the β (type II) error level); and (4) for continuous outcomes, the standard deviation of the measurements?  Sample size not adequately stated as well as what the effect size is. Cannot be replicated. | We rewrote in Line 107-111. |
|  | **3) Sequence generation (Item 8a)**  Method used to generate random allocation sequence  Does the description make it clear if the "assigned intervention is determined by a chance process and cannot be predicted"? | Thank you, we added to Line 115-116. |
|  | **4) Allocation concealment (Item 9)**  Mechanism used to implement random allocation sequence (such as sequentially numbered containers), describing any steps taken to conceal the sequence until interventions were assigned  Is it clear how the care provider enrolling participants was made ignorant of the next assignment in the sequence (different from blinding)? Possible methods can rely on centralised or "third-party" assignment (i.e., use of a central telephone randomisation system, automated assignment system, sealed containers).  Not clearly stated in the manuscript. The method of allocation is unblinded. | We explained in Line 117. |
|  | **5) Blinding (Item 11a)**  If done, who was blinded after assignment to interventions (for example, participants, care providers, those assessing outcomes)  Is it clear if (1) healthcare providers, (2) patients, and (3) outcome assessors are blinded to the intervention? General terms such as "double-blind" without further specifications should be avoided.  Open-label, but it would be good to state if person doing the analysis was blinded to the data. | We rewrote in Line 88, 185-186. |
|  | **6) Outcomes and estimation (Item 17a/b)**  For the primary outcome, results for each group, and the estimated effect size and its precision (such as 95% confidence intervals)  Is the estimated effect size and its precision (such as standard deviation or 95% confidence intervals) for each treatment arm reported? When the primary outcome is binary, both the relative effect (risk ratio, relative risk) or odds ratio) and the absolute effect (risk difference) should be reported with confidence intervals.  Just the odds ratio (95% CI) has been reported. | Yes. |
|  | **7) Harms (Items 19)**  All important harms or unintended effects in each group  Is the number of affected persons in each group, the severity grade (if relevant) and the absolute risk (e.g. frequency of incidence) reported? Are the number of serious, life threatening events and deaths reported? If no adverse event occurred this should be clearly stated.  Safety outcomes not mentioned. | We have reanalyzed the data of adverse events and presented in Table 6 with explanation in Line 246-248. |
|  | **8) Registration (Item 23)**  Registration number and name of trial registry  Is the registry and the registration number reported? If the trial was not registered, it should be explained why.  Yes, | We stated in Line 96. |
|  | **9) Protocol (Item 24)**  Where trial protocol can be accessed  Is it stated where the trial protocol can be assessed (e.g. published, supplementary file, repository, directly from author, confidential and therefore not available)?  Yes, in supplementary | Yes, we provided in supplementary. |
|  | **10) Funding (Item 25)**  Sources of funding and other support (such as supply of drugs) and role of funders  Are (1) the funding sources, and (2) the role of the funder(s) described?  Yes. | We deleted from MS according to the second reviewer’s suggestion. |
|  | **Reviewer #2:** The authors should be commended for running an overall well designed study, and writing a generally high quality paper.  Unfortunately I do have one major concern about how the ITT protocol was followed - the authors state an ITT protocol was developed, but the results are a bit unclear and seem to indicate that this was perhaps not the case? Clarification is urgently needed.  Apart from this one issue I am more than happy to recommend publication, but this issue must be adequately addressed as a matter of urgency, as it brings the major part of the results/conclusion of the paper into question. More details provided below. | Thank you very much, we rewrote in Line 172-177. |
|  | **Major comments:**  208: "There were a total of 7 participants who relapsed after quitting". This is a completely incorrect statement. You said you used an intention to treat methodology. Therefore every participant who dropped out of the study (the vast majority!) is assumed to have resumed smoking! This must be qualified very clearly! This mistake makes me wonder if your other results are genuinely calculated on an intention to treat basis? i.e. in Table 3, you say n=78 in each group but then your quit rate goes up over time - I suspect you have not included the drop-outs (as relapsers) in this data. This same comment can potentially be made for others of the reported results. | Thank you very much for this point.  We rewrote “Data analysis” for how to count the proportion of CAR and 7-day PAR (Line 178-186) and “Result” in Table 2-3.  We calculated CAR only participants who could quit smoking at Day 180, then checked back in every visit. The 7-day PAR, we count in each visit who could be abstinent.  So one out of 7 (No 43) who was in the intervention group and 2 (No16, No 132) out of 7 who were in the control group were not in PAR analysis because they were abstinent less than 7 days before the visit. The rest we analyzed them in the PAR (No64-intervention, No 123, 69 and 110—control). Only one out of 7 (no 69) who failed only at Day 30 and remained being abstinent through Day 180, so we analyzed in the CAR. |
|  | **Minor comments:**  Design: please include the $ symbol and report to 2 decimal places the currency information at lines 111 and 112  Ethical approval: failure to register the clinical trial prior to enrolment is a fairly big mistake under the current clinical trials rules, however, it is only very recently that a trial like this would have been considered a clinical trial at all. The excuse about the requirement not being mentioned "in the graduate program" is a bit confusing though (the reader doesn't know anything about your "graduate program" or how it relates to this mistake). I suggest replacing this statement with something like "The researchers were unaware of the requirement to register the clinical trial prior to the enrolment of participants, but amendments have been made to our institutional training materials to reduce the likelihood of this error occurring in the future." If possible, it may also be valuable to state that the HREC was made aware of this mistake and judged it to be low risk, or something to that effect.  Lines 130-131: Is there a reference to support the estimated drop-out rate of 15%?  Line 134: Fagerstorm => Fagerström  Line 148: The whole URL should be hyperlinked, not just the inner part.  Line 166: "usual pharmacy services on smoking cessation and medications" => It is important to state explicitly what these usual services are, since practice in various regions differs significantly. Most important is the conditions under which participants would gain access to NRT, and the frequency of counselling/assessments - I assume that this is the same as in the intervention group, but this must be made explicitly clear. It may be beneficial to instead report the common characteristics of treatments in both groups, then state how the intervention and control groups differed (presumably the only difference was access to PharmQuit). As it is currently written, it seems like the intervention group gets WAY more care than the control group but I do not think this is correct (and it would be a major design flaw if true).  Line 172-173: "Quit rate was determined by counting the number of visits with the pharmacist since quitting" this doesn't make sense.  Line 177 "User’s experience theory ": a reference should be included.  Line 186: " using the logistic regression" => "using logistic regression"  Line 186-187: "Data for participants with missing data were assumed to be the same as their last visit information." -> this must be justified. Was it the policy of the recording pharmacists to leave fields blank if they were unchanged? Missing data is not normally treated this way.  Line 187: " SPSS version 19" It's a little unusual to use such an ancient version of SPSS, but probably not of an importance given the statistical analyses are quite straightforward. | We added the symbol and reported 2 decimal places in Line 91-92.  We changed as in Line 97-99.  We added a reference (no 24) in Line 110.  We edited in Line 128.  We edited in Line 131.  We rewrote in Line 150-152.  We rewrote in Line 154-159.  We added. (Line 162)  We changed. (Line 184)  Thank you for this comment, we rewrote in Line 173-177. Most of the blank fields were because pharmacists could not follow-up with participants, that lead to missing data in the primary and secondary outcomes.  We have reviewed and consulted a few statisticians to deal with missing data. We have tried with mean substitution, regression imputation, and MICE. The three methods showed the same result when compared between groups. However, within group comparison, regress imputation and multivariate imputation by chained equations (MICE) showed nearly similar results. So, we decided to use MICE.      Our university only has SPSS version 19 with a license. |
|  | Results  Line 190-191: your completion rate makes me think your estimated drop-out rate of 15% was extremely naive? Unless you meant 15% drop-out per visit? Some follow-up with participants who were "unable to be contacted" might be useful to figure out what their reasons for dropping out were (presumably they relapsed and were no longer interested in quitting, but it would be nice to get some data).  196: "More medications were used by participants in the control group than in the intervention group" - this is clearly false when looking at the table. There is no significant difference, and there doesn't seem to be a huge clinical difference between 51% and 56%.  196-197: "FTND in the control group was lower in the high nicotine addiction group than in the intervention group" - This sentence is difficult to understand, as we have not been introduced to the concept of "high nicotine dependence" groups before now. Upon examination of the table it makes sense, but it is a clumsy sentence. | You are correct, we planned drop-out of 15% at the end of the study. But it was not as expected. We rewrote as your suggestion in Line 190-191.  Thank you, we deleted this sentence.  Thank you, we rewrote this part in Line 196-198. |
|  | Discussion:  287: "this good" => "this could"  Discussion re: other quit smoking apps fails to highlight that each study is using a very different protocol and this is likely the largest reason for the large discrepancies in outcomes. i.e. if you don't have face to face interviews drop out rates are higher etc. | Thank you, we edited and added this sentence to the discussion part in Line 281-283. |
